# Silencing of *Salmonella typhimurium* Pathogenesis: Atenolol Acquires Efficient Anti-Virulence Activities

**DOI:** 10.3390/microorganisms10101976

**Published:** 2022-10-06

**Authors:** Abrar K. Thabit, Khalid Eljaaly, Ayat Zawawi, Tarek S. Ibrahim, Ahmed G. Eissa, Samar S. Elbaramawi, Wael A. H. Hegazy, Mahmoud A. Elfaky

**Affiliations:** 1 Pharmacy Practice Department, Faculty of Pharmacy, King Abdulaziz University, Jeddah 21589, Saudi Arabia; 2 Department of Medical Laboratory Sciences, Faculty of Applied Medical Sciences, King Abdulaziz University, Jeddah 21589, Saudi Arabia; 3 Vaccines and Immunotherapy Unit, King Fahd Medical Research Center, King Abdulaziz University, Jeddah 21589, Saudi Arabia; 4 Department of Pharmaceutical Chemistry, Faculty of Pharmacy, King Abdulaziz University, Jeddah 21589, Saudi Arabia; 5 Medicinal Chemistry Department, Faculty of Pharmacy, Zagazig University, Zagazig 44519, Egypt; 6 Department of Microbiology and Immunology, Faculty of Pharmacy, Zagazig University, Zagazig 44519, Egypt; 7 Pharmacy Program, Department of Pharmaceutical Sciences, Oman College of Health Sciences, Muscat 113, Oman; 8 Department of Natural Products, Faculty of Pharmacy, King Abdulaziz University, Jeddah 21589, Saudi Arabia; 9 Centre for Artificial Intelligence in Precision Medicines, King Abdulaziz University, Jeddah 21589, Saudi Arabia

**Keywords:** atenolol, quorum sensing, *Salmonella* Typhimurium, virulence, biofilm, *E. coli*

## Abstract

The targeting of bacterial virulence is proposed as a promising approach to overcoming the bacterial resistance development to antibiotics. *Salmonella enterica* is one of the most important gut pathogens that cause a wide diversity of local and systemic illnesses. The *Salmonella* virulence is controlled by interplayed systems namely Quorum sensing (QS) and type three secretion system (T3SS). Furthermore, the *Salmonella* spy on the host cell via sensing the adrenergic hormones enhancing its virulence. The current study explores the possible anti-virulence activities of β-adrenoreceptor blocker atenolol against *S. enterica* serovar Typhimurium in vitro, in silico, and in vivo. The present findings revealed a significant atenolol ability to diminish the *S. typhimurium* biofilm formation, invasion into HeLa cells, and intracellular replication inside macrophages. Atenolol significantly downregulated the encoding genes of the T3SS-type II, QS receptor Lux analogs *sdiA*, and norepinephrine membranal sensors qseC and qseE. Moreover, atenolol significantly protected mice against *S. typhimurium*. For testing the possible mechanisms for atenolol anti-virulence activities, an in silico molecular docking study was conducted to assess the atenolol binding ability to QS receptor SdiA and norepinephrine membranal sensors QseC. Atenolol showed the ability to compete on the *S. typhimurium* targets. In conclusion, atenolol is a promising anti-virulence candidate to alleviate the *S. typhimurium* pathogenesis by targeting its QS and T3SS systems besides diminishing the eavesdropping on the host cells.

## 1. Introduction

Quorum sensing (QS) is a way that bacteria use to communicate with each other allowing specific processes to be regulated, such as virulence factors expression, biofilm formation, and secondary metabolites production in addition to stress adaptation mechanisms such as bacterial competition systems including secretion systems [1,2]. An interkingdom crosstalk was observed between bacteria and host cells, in which bacteria could sense the chemical changes in the surroundings [3,4,5]. Bacteria eavesdrop on the host cells using membrane sensors to sense neuroendocrine hormones that result in enhancing bacterial virulence [3,6,7]. The Gram-negative bacteria response to neuroendocrine stress hormones is well observed in the augmentation of virulence [3,8,9]. QS systems work in an inducer-receptor manner; as the QS receptors bind to their cognate autoinducers (AIs) which are mainly N-acetyl homoserine (AHLs) in Gram-negative bacteria [1,3,10]. Next, the formed QS receptor-AI complex binds to the bacterial chromosome upstream of the virulence genes controlling their regulation [2,11]. Fascinatingly, AIs also crosstalk with the adrenergic hormones starting the same signaling pathway in the host cells [6,8,12,13]. As a closed-loop, the AIs produced by bacteria stimulate the production of the neuroendocrine hormone in host cells that will be sensed resulting in augmenting virulence [6,8,9]. In this context, targeting QS as well as blocking the bacterial adrenergic sensors lead to attenuation of the bacterial virulence [3,14,15].

*Salmonella enterica* is a Gram-negative gut pathogen that is transmitted orofecally and causes diverse infections from local gastroenteritis to systemic enteric fevers [13,16]. *S. enterica* invasive infections either by typhoidal or non-typhoidal species are critical if not treated leading to an increase in mortality rates particularly in developing countries [13,17,18,19]. The resistance to antibiotics worsens the situation as even invasive nontyphoidal *S. enterica* serovars Typhimurium or Enteritidis infects that bloodstream causing serious illness [13,16,20,21]. Unfortunately, there is growing data that documents the increased resistance to different antibiotics even those that were known to be efficient in the treatment of *Salmonella* infections [17,21]. In addition to QS, there is accumulative evidence of the spying of *S. enterica* on host cells that results in enhancing the virulence [19,22,23,24]. Furthermore, *S. enterica* utilizes two types of type three secretion systems (T3SS) to regulate its invasion and intracellular replication [25,26]. For its pathogenesis, antibiotic resistance, and virulence behavior, *S. enterica* is gaining increasing interest.

Drug repurposing is an advantageous attractive strategy to find out new antibiotics and new anti-virulence agents [27,28]. Targeting bacterial virulence is an auspicious approach to attenuate bacteria enough to ease their eradication by the immune system. This approach does not affect the growth and hence does not stress bacteria to develop resistance [29,30,31]. Mitigation of bacterial resistance is proven as one of the promising approaches to overcome the resistance development [32,33], and there are several drugs, compounds, and natural compounds that were repurposed for this purpose [34,35,36,37,38]. Bearing in mind the crucial roles of QS in regulating bacterial virulence; targeting QS was proposed as a suitable way to guarantee a diminishing bacterial virulence [29,39]. Preventing bacterial spying on host cells could also assure mitigation of the virulence [8,12]. In this direction, our group gave more attention to investigating the anti-QS activities of adrenoreceptor blockers. Interestingly, several α- and β-adrenoreceptor blockers showed significant in silico, in vitro and in vivo anti-QS and anti-virulence activities [14,15]. Among the in silico screened β-adrenoreceptor blockers, atenolol showed a marked ability to compete on different QS receptors [15]. Atenolol is used primarily to treat high blood pressure and heart-associated chest pain. Other uses include the prevention of migraines and treatment of certain irregular heartbeats [40,41]. In the current study, the anti-QS activities and effects of atenolol were explored prior to its application as an anti-virulence agent adjuvant to antibiotics. Furthermore, the atenolol effects on T3SS invasion and intracellular functions were evaluated.

## 2. Materials and Methods

### 2.1. Bacterial Strains and Growth Conditions

*Escherichia coli* K-12 MG1655 and *Salmonella enterica* serovar Typhimurium (NCTC 12023) were used in this work. Fresh overnight cultures of *E. coli* K-12 were cultivated in AB minimal media [42] complemented with glucose (0.5%) and 2.5 mg/mL thiamine [43]. Fresh overnight cultures of *S. typhimurium* were cultivated in Luria-Bertani (LB) broth or Tryptic Soy Broth (TSB) provided with 0.001 μM AHL [12,19]. The cultures were adjusted to cell density 1 × 10^6^ CFU/mL (OD600 = 0.4) before each experiment.

### 2.2. Chemicals and Microbiological Media

All the chemicals used in this study were of pharmaceutical grade. The microbiological media were ordered from Oxoid (Hampshire, UK). Atenolol (CAS number: 29122-68-7), N-hexanoyl-DL-homoserine lactone (AHL) (CAS Number: 106983-28-2), thiamine (CAS number: 67-03-8), DL-norepinephrine hydrochloride (CAS Number: 55-27-6), and Dulbecco’s Modified Eagle’s Medium (DMEM) medium were obtained from Sigma-Aldrich (St. Louis, MO, USA). It is worthy to mention that used atenolol was in form of hydrochloride salt and it was dissolved in water and kept in stocks prior to use in all the next experiments.

### 2.3. Determination of Minimum Inhibitory Concentrations (MICs) and Atenolol Effect on Bacterial Growth

The broth microdilution method was used to determine the atenolol MICs against *E. coli* K-12 and *S. typhimurium* according to the Clinical Laboratory and Standards Institute Guidelines (CLSI, 2020) [44].

The effect of atenolol at sub-MIC (1/5 MIC) on bacterial growth was evaluated as previously described [31,45]. Briefly, the bacterial viable counts of cultures provided or not with atenolol at 1/5 MIC at different time points were performed.

### 2.4. Evaluation of Adhesion and Biofilm Formation

*E. coli* K-12 and *S. typhimurium* were cultivated in the growth conditions mentioned above before assaying the bacterial adhesion and the biofilm formation in the presence or absence of atenolol at sub-MIC. As described previously, the absorbances of crystal violet staining adhered or biofilm forming bacterial cells in treated or untreated cultures after 1 h or 24 h were used to assess the effect on adhesion and biofilm formation [13,46]. Briefly, Overnight cultures were cultivated, diluted with tryptone soya broth (TSB) and the optical densities were adjusted to a cell density of 1 × 10^6^ CFU/mL, 200 μL aliquots of the prepared bacterial suspensions were transferred into sterile 96-well polystyrene microplates and incubated at 37 °C overnight. The non-adherent cells were washed out and the, while the adherent cells were fixed with 99% methanol for 30 min, and stained with 1% crystal violet for 20 min. The unattached stain was washed off, and the plates were left to dry. The crystal violet dye was extracted by 33% glacial acetic and the optical densities were measured at 590 nm. The Atenolol inhibitory effect on biofilm formation was visualized by allowing the formation of biofilm coverslips in the presence or absence of atenolol at sub-MIC, as described before [11,12].

### 2.5. Evaluation of S. typhimurium Invasion and Intracellular Replication

The *S. typhimurium* internalization within HeLa and macrophage cells in the presence or absence of atenolol at sub-MIC was performed by gentamicin protection assay [12,19,47]. The *S. typhimurium* cultures were grown in the presence or absence of atenolol at sub-MIC in the conditions described above in the presence of 0.001 μM AHL, and a master mix (1 × 10^5^ bacteria/well) with multiplicity of infection (MOI 1) was prepared in DMEM in 24-well plates. For invasion and intracellular replication assays, HeLa cells (5 × 10^5^ cells/well) or RAW264.7 (2 × 10^5^ cells/well) were used for seeding the DMEM medium. After half hour, the wells were washed with pre-warmed phosphate buffer saline (PBS). The gentamycin at 100 μg/mL was added for 1hr to kill the extracellular adhered bacterial cells. For invasion assay, HeLa cells were lysed with 0.1% Triton X-100 at 25 °C for 25 min. Viable counts for inoculum and lysates were performed. For evaluation of the intracellular replication, the infected macrophages were washed with PBS and lysed after 2 and 16 h with 0.1% TritonX-100 for 25 min. The viable counts were performed for inoculum and lysates, and the phagocytosed cell numbers/relative untaken cells (2 h against inoculum) and x-fold intracellular replication (16 h against 2 h) was calculated.

The *Salmonella*-infected HeLa or macrophages were immunostained as described earlier (3, 14, 23). The cell lines infection was performed as described above on cover slips and then Infected cells were fixed with 2% paraformaldehyde for 25 min. Bovine serum albumin (BSA) (2%) was used as a blocking solution for 1 h. Rabbit anti-Salmonella O antigen (Difco, BD; San Joes, CA, USA) were added to fixed cells for 3 h. Next, anti-rabbit tagged with a green fluorescent protein (GFP) (Abcam; Eugene, OR, USA) was added, and the mixture was left for 1 h. The macrophages were counter-stained with blue fluorescent diamidino-2-phenylindole dye (DAPI) (Thermo Fisher Scientific; Bothell, WA, USA) for 1hr. A confocal laser scanning microscope LSM780 (Carl Zeiss, Jena, Germany) was used to take photos.

### 2.6. Quantitative RT-PCR

The RNA of *S. typhimurium* treated or not with atenolol at sub-MIC was obtained as before described [12]. The cDNA was synthesized using a high-capacity cDNA reverse transcriptase kit (Applied Biosystem, San Francisco, CA, USA). Next, cDNA was amplified with the Syber Green I PCR Master Kit (Fermentas, Maryland, USA) in a multi-well plate using the Step One instrument (Applied Biosystem, USA). The relative expressions were calculated by the comparative threshold cycle (^∆∆^Ct) method [48]. The used primers are listed in Table 1.

### 2.7. In Vivo Anti-Virulence Activity

To assess the atenolol in vivo anti-virulence activity against *S. typhimurium*, the mice survival model was performed as formerly shown [12,19]. The Ethical Committee of the at Faculty of Pharmacy, King Abdulaziz University, Jeddah, Saudi Arabia approved the protocol of the in vivo experiments in this study. The experiments were conducted in compliance with according to guidelines of the declaration of Helsinki (Code # PH-1442-13).

Briefly, *S. typhimurium* cultures were overnight cultured with atenolol at sub-MIC or norepinephrine (50 μg/mL) at 37 °C and then about 1 × 10^6^ CFU/mL bacterial cells were suspended in PBS. Five mice groups were assigned, each comprises 10 female healthy (3-weeks old) mice. The first and second groups were either not injected or intraperitoneally injected with sterile PBS. The other three groups were intraperitoneally injected with 100 μL of untreated *S. typhimurium* or *S. typhimurium* treated with atenolol at sub-MIC or norepinephrine (50 μg/mL). The experiment was performed over 5 days and mice survival was plotted by Kaplan–Meier method.

### 2.8. Molecular in Silico Studies

#### 2.8.1. Ligand and Protein Preparations

The 3D structure of atenolol was built on the MOE Builder within the Molecular Operating Environment (MOE 2019.012) using the SMILES obtained from the PubChem database (https://pubchem.ncbi.nlm.nih.gov/) (accessed on 24 May 2022). The ligand was energy minimized and protonated at physiological pH 7.4.

The crystal structures of the target proteins (PDB ID: 4LFU, and 3JZ3 for *E. coli* SdiA, and *E. coli* QseC, respectively) were downloaded from the RCSB Protein Data Bank (https://www.rcsb.org/ accessed on 4 May 2022). The proteins were prepared for docking using a QuickPrep panel to minimize energy, protonate, fix, and tether atoms along with deleting unnecessary water molecules.

#### 2.8.2. Docking Experiments

The binding pocket of the targets was determined by the MOE Site Finder and the position of the co-crystallized ligand. The Computed Atlas for Surface Topography of Proteins (CASTp; http://sts.bioe.uic.edu/castp/index.html, accessed on 28 May 2022) was used to calculate the pocket area/volume of the proteins [51]. Re-docking of the co-crystallized ligand was performed to validate the use of the protein in structure-based drug design.

Docking for the ligands in the active site was performed using Molecular Operating Environment (MOE) 2019.0102 with Alpha triangle placement, Amber10:EHT forcefield, refinement with forcefield and scoring using Affinity dG as the default settings.

### 2.9. Statistical Analysis

The statistical significance was examined by the student’s *t*-test (unless mentioned), where a *p* value < 0.05 is considered significant (GraphPad Prism Software, v.8, San Diego, CA, USA). The experiments including effect on bacterial growth, biofilm formation, internalization in HeLa cells or macrophages and quantification of the expression of tested genes were conducted in triplicate, and the data are presented as the means ± standard error.

## 3. Results

### 3.1. Determination of MICs and Its Effect on Bacterial Growth

The MICs were considered the lowest atenolol concentration that can inhibit bacterial growth. Atenolol inhibited the growth of both *S. typhimurium* and *E. coli* K-12 at 2 mg/mL.

**Figure 1:**
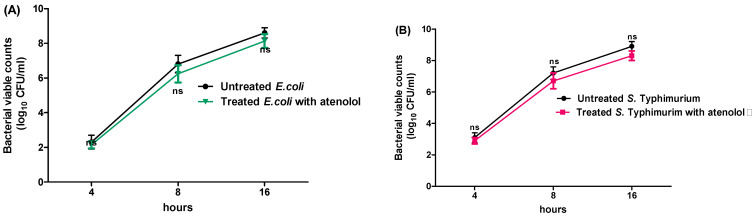
Effect of atenolol at sub-MIC on bacterial growth. (**A**) *E. coli* K-12, and (**B**) *S. typhimurium* were overnight cultured in the presence or absence of atenolol. There was no significant effect of atenolol on the growth. The experiment was performed in triplicate and results are expressed as the means ± standard deviation with *p* value < 0.05 considered statistically significant.

To exclude the effect of atenolol on bacterial growth, the antivirulence activities were evaluated at sub-MIC (1/5 MIC). For further testing, the bacterial growth was evaluated in the presence of atenolol at sub-MIC and compared with the growth of untreated bacterial cultures. There were no significant differences between viable counts of *S. typhimurium* and *E. coli* K-12 in the presence or absence of atenolol at sub-MIC (Figure 1).

### 3.2. Effect on Bacterial Adhesion and Biofilm Formation

**Figure 2:**
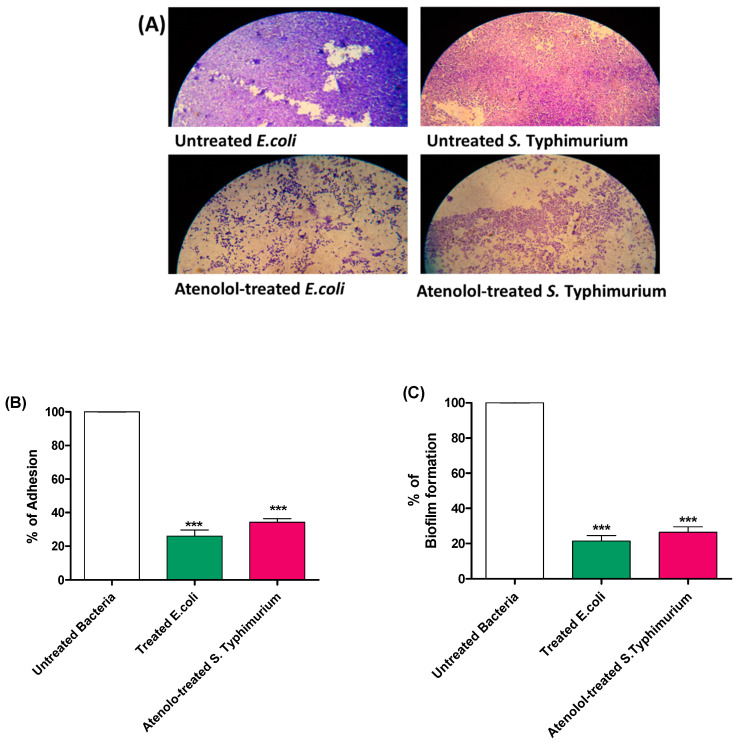
Effect of atenolol at sub-MIC on bacterial adhesion and biofilm. (**A**) Representative light microscope images show the obvious reduction in the *S. typhimurium* and *E. coli* K-12 biofilm formation in the presence of atenolol. The absorbances of crystal violet staining (**B**) Adhered cells, and (**C**) Biofilm forming cells in the presence of atenolol compared to untreated control. Atenolol significantly diminished the *S. typhimurium* and *E. coli* K-12 adhesion and biofilm formation (*** *p* < 0.0001). The experiment was performed in triplicate and results are expressed as the means ± standard deviation with *p* value < 0.05 considered statistically significant.

The inhibitory effect of atenolol on *S. typhimurium* and *E. coli* K-12 adhesion and biofilm formation was assessed using the crystal violet method. The adhered or biofilm forming bacterial cells were stained with crystal violet after 2 h or 24 h, respectively. Captured light microscope images of formed biofilms on cover slips showed the marked effect of atenolol on diminishing the biofilm formation (Figure 2A). The absorbances of the extracted crystal violet staining the bacterial cells treated with atenolol were photometrically measured and compared to the absorbances of untreated cultures. Atenolol significantly decreased bacterial adhesion and biofilm formation; the data are presented as percentage changes from untreated control (Figure 2B).

### 3.3. Effect on S. typhimurium Invasion and Internalization

**Figure 3:**
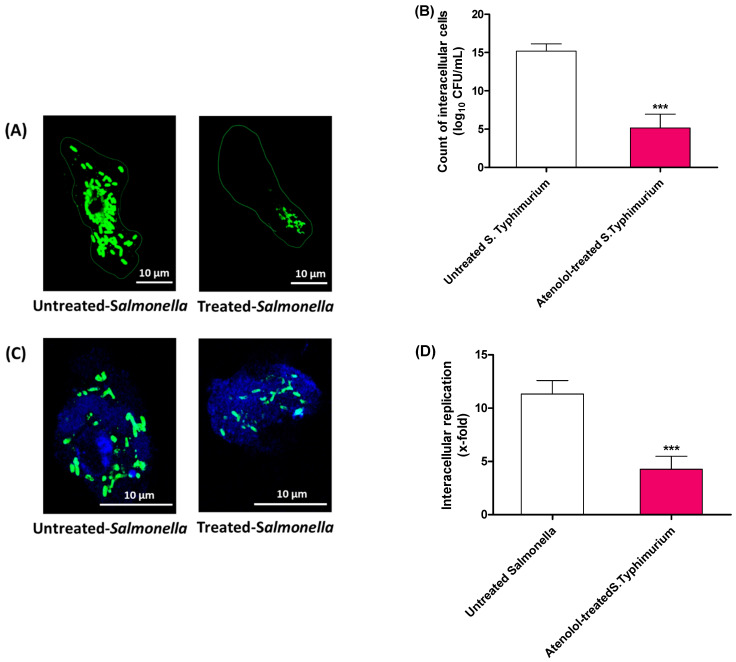
Effect of atenolol at sub-MIC on *S. typhimurium* invasion and intracellular replication. (**A**) A representative fluorescent microscopic image shows the obvious mitigation of the *S. typhimurium* invasion to HeLa cells in the presence of atenolol. (**B**) The viable count of the invading *S. typhimurium* to HeLa cells in the presence and absence of atenolol. Atenolol significantly attenuated *S. typhimurium*, and the numbers of invading cells significantly decreased. (**C**) A representative fluorescent microscopic image shows the marked diminishing of the intracellularly replicating *S. typhimurium* in raw macrophages in the presence of atenolol. (**D**) The intracellular replicating *S. typhimurium* counted against the initial invading bacterial cells and the fold changes were calculated. Atenolol significantly decreased the intracellular replication of *S. typhimurium* (*** *p* < 0.0001). The experiment was performed in triplicate and results are expressed as the means ± standard deviation with *p* value < 0.05 considered statistically significant.

The antivirulence activity of atenolol at sub-MIC on *S. typhimurium* invasion and intracellular replication was evaluated by employing a gentamicin protection assay. Significantly, atenolol attenuated the invasiveness of *S. typhimurium* in HeLa cells (Figure 3A,B) and also diminished the numbers of intracellularly replicated bacterial cells inside the raw macrophage (Figure 3C,D).

### 3.4. Effect on the Expression of Virulence Encoding Genes

**Figure 4:**
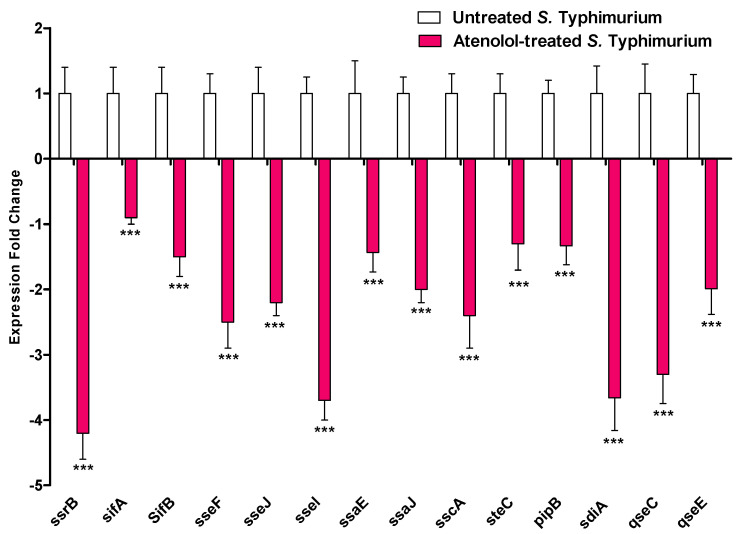
Effect of atenolol at sub-MIC on the expression of *S. typhimurium* virulence encoding genes. The expression of the QS encoding gene *sdiA* and the norepinephrine membranal sensor encoding genes, *qseC* and *qseE*, and TTSS encoding genes *ssrB*, sifA, sifB, sseF, sseJ. sseI, ssaE, ssaJ, *sscA, steC*, and *pipB* were normalized to *gyrB* housekeeping gene in the presence of atenolol in comparison to untreated controls. Atenolol significantly downregulated the expression of *S. typhimurium* virulence encoding genes (*** *p* < 0.0001). The experiment was performed in triplicate and results are expressed as the means ± standard deviation with *p* value < 0.05 considered statistically significant.

The expressions of the encoding genes of different virulence factors in *S. typhimurium* were quantified with RT-PCR in the presence of atenolol at sub-MIC and compared to untreated controls. The expression of TTSS-II, *sdiA* QS receptor, and sensor kinases *qseC* and *qseE* encoding genes were determined in the presence or absence of atenolol. Interestingly, atenolol showed a significant ability to downregulate the tested genes when the genes’ expressions were normalized to *gyrB* gene (Figure 4). For further confirmation the tested genes’ expressions were normalized to another housekeeping gene 16S rRNA. Atenolol significantly downregulated the expression of tested genes (Figure S1).

### 3.5. The Effect on the In Vivo Pathogenesis

**Figure 5:**
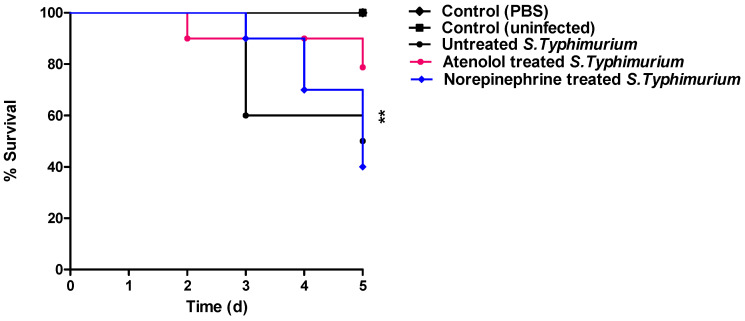
Atenolol diminished the *S. typhimurium* virulence. However, all the mice survived in the negative control groups, and only 50% of mice survived in the positive control mice group injected with untreated *S. typhimurium*. Furthermore, norepinephrine increased the *S. typhimurium* pathogenesis causing a death rate of 60%. On the other hand, atenolol protected mice and decreased death rate to 20%, which indicates the significant atenolol ability to reduce the *S. typhimurium* capacity to kill mice (log rank test *p* = 0.0034). Mice survival in each group was recorded every day over 5 days, plotted using the Kaplan–Meier method and significance (*p* < 0.05) was calculated using the Log rank test. ** *p* < 0.01.

In vivo mice protection assay was employed to test antivirulence activity of atenolol at sub-MIC. For more convincing, a mice group was injected with norepinephrine. All mice groups that were injected with PBS or kept un-injected survived. Meanwhile, only five out of ten mice survived in the mice group injected with untreated *S. typhimurium*. Interestingly, norepinephrine enhanced the *S. typhimurium* pathogenesis, six deaths out of ten (60%) were recorded. On the other hand, atenolol significantly protected mice against *S. typhimurium* pathogenesis, recording only 2 deaths out of ten (20%) (log rank test for trend *p* = 0.0034) (Figure 5).

### 3.6. Molecular Docking In Silico Study

#### 3.6.1. Atenolol Binding on SdiA and QseC

A two-step docking protocol, consisting of a preliminary rigid receptor approach and a further induced-fit docking, was applied to explore the interactions of atenolol with bacterial targets regulating virulence genes; the *E. coli* SdiA (PDB ID: 4LFU), and the *E. coli* QseC receptor (PDB ID: 3JZ3).

The protein structure of *E. coli* SdiA which has been solved at 2.26 Å is composed of a ligand-binding domain (LBD) represented by residues 5–167 at the N-terminus and a DNA-binding domain (DBD) represented by residues 184–240 located at the C-terminus. Both of them are connected together by a 16 amino acid linker. The topology of the ligand-binding domain is basically a sandwich of a central antiparallel five-stranded β-sheet in between two α-helices. The ligand-binding site with the co-crystallized tetraethylene glycol molecule can be found as a concave surface on the β-sheet. The binding pockets of the biotargets were defined by matching the position of the co-crystallized ligand with the sites obtained from MOE Site Finder.

**Figure 6:**
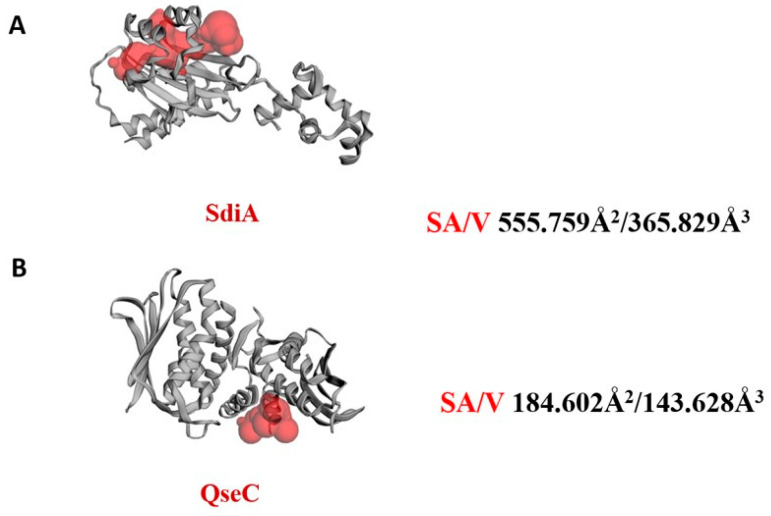
Cartoon representation of the binding site topology at the bacterial targets (**A**) SdiA, (**B**) QseC. Putative pockets; red color, were calculated via the on-line Computed Atlas of Surface Topography of proteins (CASTp; http://sts.bioe.uic.edu/castp/index.html, accessed on 10 June 2022).

The *E. coli* QseC contains a total of 310 amino acids divided into two chains, with chain B more structured than chain A in the 2.5 Å crystal structure. The final structure of which carries a short N-terminal β strand (β1) and an α helix (α1), and the catalytic domain. The *E. coli* QseC had no co-crystallized ligand, and the active site selection depended upon MOE Site Finder and CASTp predictions. The 3D protein structure and the putative binding pockets with the calculated Richards’ solvent accessible surface area (SA) and volume (V) of the biotargets as estimated using the Computed Atlas for Surface Topography of Proteins (CASTp) (Figure 6).

#### 3.6.2. Docking Simulations on *E. coli* SdiA QS Receptor

Docking of atenolol in *E. coli* SdiA resulted in generally comparable binding position, score, and interactions with the co-crystallized ligand (TEG) which was re-docked for validation and comparison as summarized in Table 2. The simply root-mean-square deviation (RMSD) for docking results of the co-crystallized was below 2.00 Å; indicating a valid and reliable docking procedure. Atenolol showed −6.4145 kcal/mol score which is slightly lower than that of the co-crystallized ligand TEG; −6.1143 Kcal/mol, indicating a good binding affinity of the ligand.

**Figure 7:**
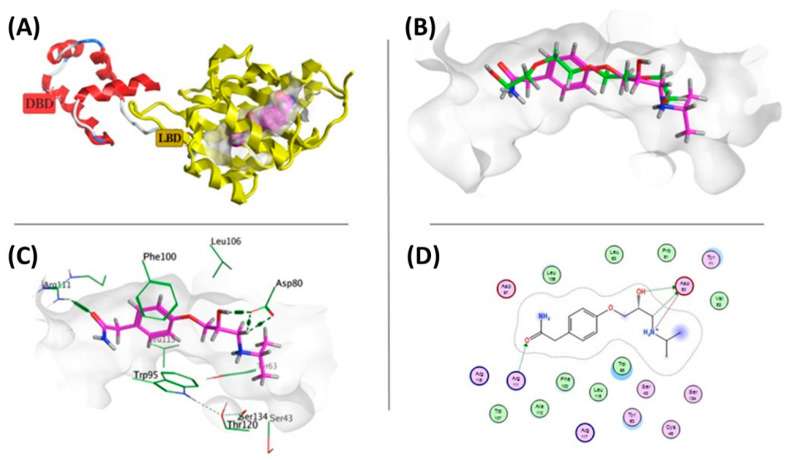
(**A**) Three-dimension cartoon representation of *E. coli* SdiA; Ligand-Binding Domain (LBD) is in yellow color and DNA-Binding Domain is in red color (**B**) Superimposition of TEG and atenolol in the molecular surface of the active site; Atenolol is represented in purple and TEG in green. (**C**) Three-dimension Atenolol- *E. coli* SdiA interaction diagram. Atenolol is in thick purple sticks within the molecular surface of the active site, amino acid residues of the active site are shown as thin green sticks. H-bond is presented as green dots. (**D**) Two-dimension Atenolol- *E. coli* SdiA interaction plot.

Both atenolol and TEG exhibited numerous hydrophobic interactions with Tyr63, Tyr71, Trp95, Phe100, Leu106, Trp107, Ala110, Arg111, Leu115, Arg116 and Arg117 amino acid residues, representing the hydrophobic lining of the binding pocket. The acceptor oxygen of amide group in atenolol formed a hydrogen bond with the basic Arg111 and the hydroxyl group interacted with Asp80 through H-bond formation. Moreover, a protonated amino group formed an ionic bond with Asp80 (Figure 7).

#### 3.6.3. Docking Simulations on *E. coli* QseC Receptor

QseC autophosphorylation occurs in response to epinephrine/norepinephrine (EPI/NEI) detection as a part of a complex pathway [9]. Norepinephrine (NE) was used in the docking process of atenolol into QseC for comparison as a reference compound as there was no co-crystallized ligand. Docking score for atenolol was −5.7045 Kcal/mol, while for norepinephrine was −4.4487. This suggests that atenolol is better than norepinephrine in binding affinity towards the active site pocket. Details of the docking results are illustrated in Table 3.

**Figure 8:**
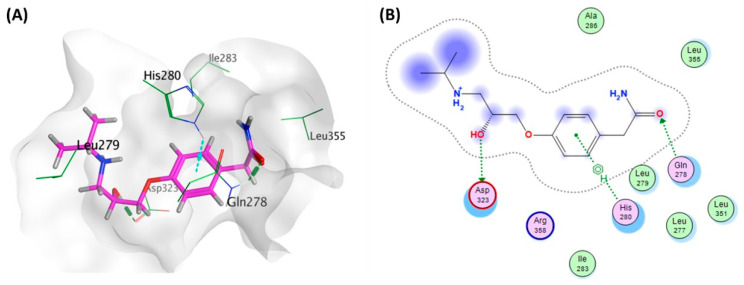
(**A**) 3D Atenolol- *E. coli* QseC interaction diagram, Atenolol is in thick purple sticks within the molecular surface of the active site, amino acid residues of the active site are shown as thin green sticks. H-bond is presented as green dots and pi-H bond is presented as turquoise bonds. (**B**) 2D Atenolol- *E. coli* QseC interaction plot.

Several key amino acid residues played a pivotal role in anchoring atenolol within the *E. coli* QseC pocket. Amidic and hydroxyl groups showed hydrogen bond interactions with Gln278 and Asp323, respectively, as well as pi-H interaction of phenyl ring with His280. In addition, atenolol formed hydrophobic contacts with Gln287, Leu279, His280, Ile283, Asp323, Leu351 and Leu355 amino acid residues as shown in Figure 8.

## 4. Discussion

Antibiotic misuse is considered one of the main causes of the development of bacterial resistance. The decreased numbers of newly discovered efficient antibiotics cannot resolve this issue and bacterial resistance development is augmented to be one of the major public health problems [52,53]. In this context, several approaches have been proposed; targeting bacterial resistance is a promising one [16,31,54,55]. This approach confers anti-virulence agents that can be used in addition to antibiotics in particular in aggressive resistant bacterial infections [2,56]. The main concept of this approach is weakening bacterial virulence to be easily killed by host immune cells, which occurs without affecting growth and hence does not stress them to develop resistance [10,29]. The potent anti-virulence activities of several chemical compounds, drugs, and natural products proved the legibility of this approach [10,14,15]. To exclude any effect of atenolol on bacterial growth, all the performed experiments were performed using sub-MIC concentration. Moreover, the effect of atenolol at sub-MIC was evaluated on bacterial growth. It was shown that atenolol at sub-MIC did not have any significant effect on bacterial survival.

In addition to its clinical importance, *Salmonella* recruits several interplayed systems that control its pathogenesis [18,24]. These systems control the overall virulence behavior of *Salmonella* and their targeting could guarantee its virulence mitigation. The most important system is quorum sensing (QS) which is the key regulator of the production of *Salmonella* virulence factors, motility, and biofilm formation [22,57,58]. Indeed, *Salmonella* utilizes Lux-analogs SdiA to sense a variety of AHLs autoinducers [19,22,59]. The QS roles are very important for *Salmonella* adhesion and biofilm formation, as the *sdiA* mutants were not able to adhere to abiotic surfaces and did not form strong biofilms similar to those formed by wild-type *Salmonella* [19]. Meanwhile, atenolol virtually showed a marked ability to compete on SdiA, as it decreased the expression of its encoding gene. Furthermore, atenolol significantly decreased the *E. coli* and *S. typhimurium* adhesion and biofilm formation.

In addition to the main roles of QS in controlling the production of virulence factors, QS activates the expression of different secretion systems including the T3SS [60,61]. *Salmonella* employs two T3SS injectosomes to regulate its invasion into the host cells and intracellular replication inside immune cells [18,26]. The arrangement of several virulence genes in specific loci on the bacterial chromosome is called pathogenicity islands. To our knowledge, the virulence genes are arranged on 22 *Salmonella* pathogenicity islands (SPIs) [62]. Particularly, SPI1 and SPI2 encode the two types of T3SS; the first type T3SS-SPI1translocates its effectors in the early stages to modify the cytoskeleton modulating the *Salmonella* invasion into the host cells. Later, when *Salmonella* is engulfed inside the immune cell’s phagosomes, T3SS-SPI2 works in the surrounding drastic conditions translocating its effectors to the cytoplasm to ensure the *Salmonella* survival and even replication in the phagosomes [25,46]. Interestingly, atenolol downregulated the expression of the genes encoding the T3SS-SPI2 structure, function and regulation (Table 2). It was shown that the *Salmonella sdiA* mutants were found to be deficient in invasion and intracellular replication in comparison to wild-type [19]. Bearing in mind the significant anti-QS atenolol activity that in turn diminishes the expression of T3SS in addition to decreasing the expression of its encoding genes, it can be said that atenolol could interfere with the T3SS. For attesting to these findings, the effect of atenolol at sub-MIC on the *Salmonella* invasion into HeLa cells and intracellular replication inside macrophages was investigated. In compliance with interference with T3SS, atenolol significantly diminished the *Salmonella* invasion and decreased its replication in macrophages.

While bacteria can communicate with each other using chemical signals as known for QS, they can inter-species talk. For instance, *Salmonella* SdiA QS receptor can sense autoinducers of another species [5,19]. More interestingly, bacteria can hear our stress as bacteria can sense the adrenergic hormones using special sensors on their membranes [7,9]. The adrenergic hormones crosstalk with the autoinducers of QS activating the same signaling pathway, and enhancing the virulence [6,8,9]. Parallelly, autoinducers stimulate the norepinephrine surge in host cells resulting in augmenting the virulence [7,8]. The bacterial eavesdrop is observed mainly among the gut pathogen; *E. coli* K-12 as well as *Salmonella* spy on the host cells using membranal sensors QseC and QseE [3,6,8,9]. The β-adrenoceptor blockers could counteract the norepinephrine compromising effect on the host defense [5]. Atenolol showed considered ability to compete with norepinephrine on QseC, and significantly decreased the expression of norepinephrine sensors encoding genes *qseC* and q*seE*. These results indicate the possible atenolol’s diminishing effects on *Salmonella* espionage on host cells resulting in mitigation of bacterial virulence.

To sum up the anti-virulence effects of atenolol, a mice protection assay was performed against *S. typhimurium*. Atenolol at sub-MIC significantly mitigated the *Salmonella* virulence and decreased its capacity to kill mice. The current data document the potent anti-virulence activities of atenolol via targeting QS, interfering with T3SS and diminishing bacterial espionage. However, further detailed pharmacological studies have to be conducted to avoid any predicted adverse effects.

## 5. Conclusions

*S. enterica* is a clinically important pathogen and develops resistance to antibiotics. The drug repurposing approach for attenuating bacterial virulence is supposed to be an efficient approach to overcome resistance development. In the current study, atenolol was evaluated in silico, in vitro and in vivo to be repositioned as an anti-virulence adjuvant to antibiotics. Atenolol significantly diminished *Salmonella* adhesion, biofilm formation, invasion and intracellular replication. Atenolol downregulated the T3SS encoding genes and protected mice against *Salmonella*. Furthermore, it showed the ability to bind to Qs receptor SdiA and norepinephrine sensors QseC and QseE, in addition to down regulation of the encoding genes. These findings suppose the atenolol as a potent anti-virulence candidate to be used besides antibiotics in the treatment of resistant infections. Future clinical studies are warranted to confirm findings from this study in the treatment of infections caused by *Salmonella* spp.

## Figures and Tables

**Table 1:** The primers used in this study.

Target Gene	Primer Sequence: 5′-3′	Gene Significance	Reference
***gyrB***	F: GTGATCAGCGTCGCCACT R: GCGCGGTGATCAGCGTC	Housekeeping	[13]
**16S rRNA**	F: CGGGGAGGAAGGTGTTGTG R: GAGCCCGGGGATTTCACATC	Housekeeping	[49]
***sdiA***	F: AAT ATC GCT TCG TAC CAC R: GTA GGT AAA CGA GGA GCA G	Adhesion	[50]
***qseC***	F: GGTACCAAATTGACGCAACGTCTCAG R: GAATTCGCCCAACTTACTACGGCCTC	Sensor to adrenergic hormones	[7,12]
***qseE***	F: GGTACCAGCGACACGTTGAAGCGC R: GAATTCGCGTGTTTGTCAGATGCAGG	Sensor to adrenergic hormones	[7,12]
***ssrB***	F: CGCAGGTGCTAATGGCTATG R: TTTGCAATGCCGCTAACAGA	SPI2-expression regulation	[13]
***ssaE***	F: CCGCAGCAATATCAGCAAAA R: AAGTGCGCTGTTATGGTAACGA	SPI2-intracellular replication	[13]
***ssaJ***	F: TGTCGAGCAGTCGCAGTTTATTA R: TGCCTATGCGGATAACCGTTA	SPI2-intracellular replication	[13]
***sseF***	F: TCAGGAATCGCTATTTCTATG R: GTCAGGCTAACGGAGGTAA	SPI2-intracellular replication	[13]
***sseJ***	F: AATAAATCACATCCCAAGC R: ACTCAGTCCAGGTAAATCC	SPI2-intracellular replication	[13]
***sseI***	F: GATACCCCCCCTGAAATGAGTT R: GTGACAAATCGTCCAGATGCA	SPI2-intracellular replication	[13]
***sifA***	F: TACCACCACCGCATACCCA R: ACGAGGAACGCCTGAAACG	*Salmonella*-inducing filaments (SPI2)	[13]
***sifB***	F: TGATACTCAGCCTGCCCAC R: GCTCAGGGAACAAGCAAC	*Salmonella*-inducing filaments (SPI2)	[13]
***sscA***	F: GGCTCGCTGCGTATGTTGTT R: GCCGGCGAATTCTTTTACCT	SPI2 chaperon intracellular replication	[13]

**Table 2:** Docking results for both TEG and atenolol with the *E. coli* SdiA QS receptor.

Ligand	Rigid Receptor Protocol		Induced-Fit Protocol		H-Bond Interactions	Hydrophobic Interactions	*pi*-Interactions
	S Score Kcal/mol	RMSD	S Score Kcal/mol	RMSD			
Atenolol	−6.2580	1.6417	−6.4145	1.05195	Asp80, Arg111 In addition to the ionic bond with Asp80	Tyr63, Tyr71, Trp95, Phe100, Leu106, Trp107, Ala110, Arg111, Leu115 and Arg116,	-
TEG	−6.1462	0.8057	−6.1143	0.8514	Asp80, Arg111 and Asp97	Tyr71, Trp95, Phe100, Leu106, Trp107, Ala110, Arg111, Leu115 and Arg116,	Phe100 (H-*pi*)

RMSD, simply root-mean-square deviation.

**Table 3:** Docking results for both norepinephrine and atenolol with *E. coli* QseC receptor.

Ligand	Rigid Receptor Protocol		Induced-Fit Protocol		H-Bond Interactions	Hydrophobic Interactions	*pi*-Interactions
	S Score Kcal/mol	RMSD	S Score Kcal/mol	RMSD			
Atenolol	−4.9760	**2.0503**	−5.7045	1.0910	Gln278, and Asp323	Gln278, Leu279, His280, Ile283, Asp323, Leu351 and Leu355.	His280 (*pi*-H)
NE	−4.4469	1.8588	−4.4487	1.6905	Ser319, Ser320, Ser354 and Asp323.	Gln278, His280 Ser319, Asp323, Leu351 and Ser354,	-

RMSD, simply root-mean-square deviation.

## Data Availability

All data included in the main text.

## Supplementary Materials

The following supporting information can be downloaded at: https://www.mdpi.com/article/10.3390/microorganisms10101976/s1, Figure S1: Effect of atenolol at sub-MIC on the expression of *S. typhimurium* virulence encoding genes.
