# Supplementary material for: Silencing of Salmonella typhimurium Pathogenesis: Atenolol Acquires Efficient Anti-Virulence Activities
Source: Microorganisms. 2022 Oct 6;10(10):1976. doi: 10.3390/microorganisms10101976 (PMC9612049; doi:10.3390/microorganisms10101976)
Supplement: Supplementary file 1 [file microorganisms-10-01976-s001.zip › microorganisms-1901590-supplementary.pdf]

Supplementary Data

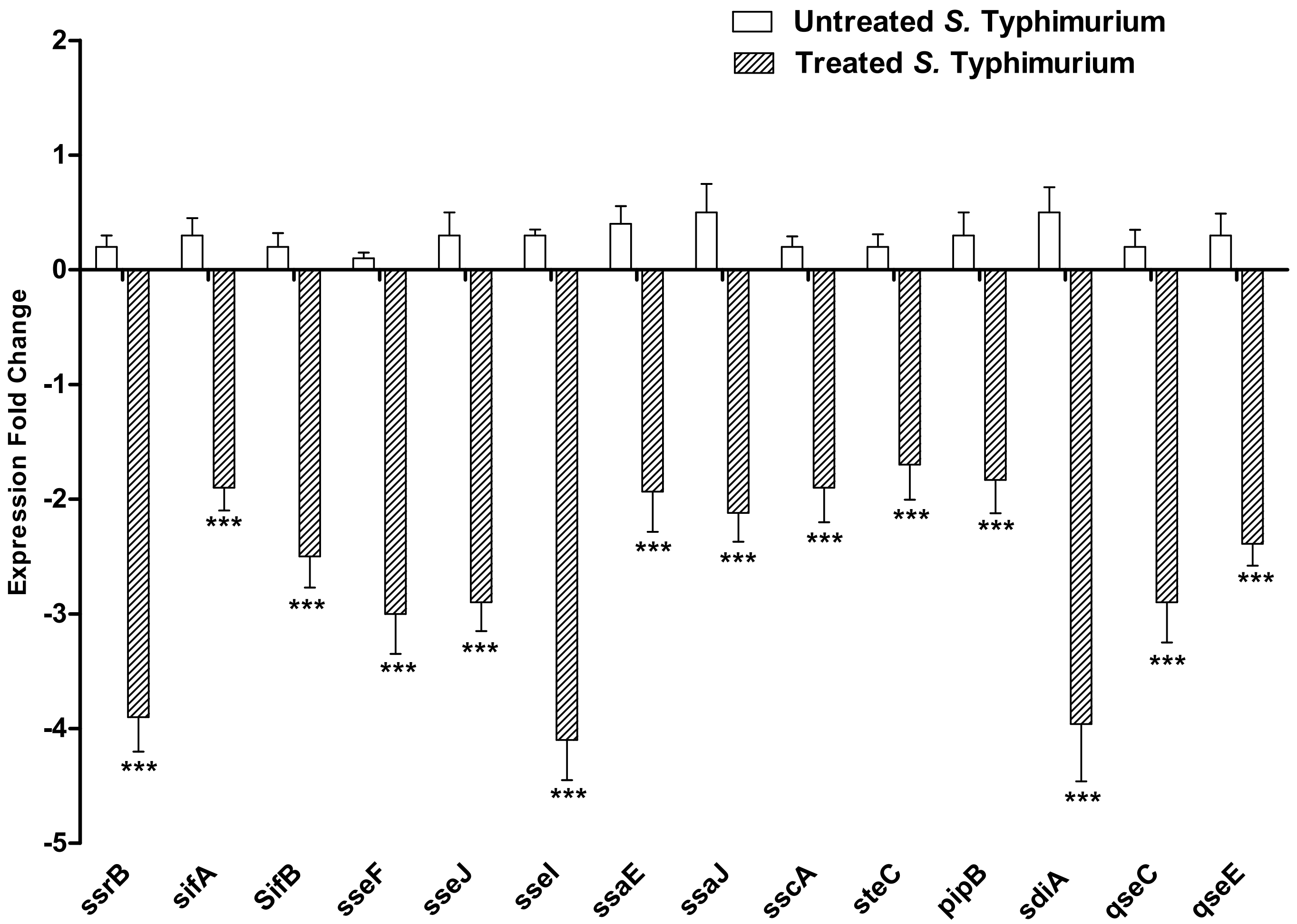

Figure S1. Effect of atenolol at sub-MIC on the expression of *S.* Typhimurium virulence encoding genes. The expression of tested genes was normalized to 16s rRNA housekeeping gene. Atenolol significantly downregulated the expression of S. Typhimurium virulence encoding genes (*** $p < 0.0001$).
